# Scanning faces: a deep learning approach to studying eye movements in prosopagnosia

**DOI:** 10.3389/fneur.2025.1616509

**Published:** 2025-09-10

**Authors:** Atlas Kazemian, Ipek Oruc, Jason J. S. Barton

**Affiliations:** ^1^Department of Ophthalmology and Visual Sciences, University of British Columbia, Vancouver, BC, Canada; ^2^Department of Medicine (Neurology), University of British Columbia, Vancouver, BC, Canada; ^3^Department of Psychology, University of British Columbia, Vancouver, BC, Canada

**Keywords:** face recognition, scanpath, artificial intelligence, neural network, developmental

## Abstract

**Background:**

Healthy individuals show fixation biases when scanning faces, likely toward the regions that are most informative for identifying faces. Some reports suggest that subjects with prosopagnosia, an impairment in face recognition, have anomalous face scanning.

**Objective:**

Our goal was to determine whether an approach using artificial intelligence could identify key scanning markers of prosopagnosia.

**Methods:**

We used an image-classification technique based on deep learning to study the fixations of subjects with and without prosopagnosia during a face recognition task. We identified the number of fixations that maximizes classification performance and developed two methods of displaying scanpaths as images, each used to train a convolutional neural network.

**Results:**

Optimal classification of acquired prosopagnosic from control trials required four fixations, with an AUC of 80%. The model showed a greater tendency to fixate the lower face and the right eye in acquired prosopagnosia. Optimal classification of developmental prosopagnosic from control trials required 16 fixations, with an AUC of 69%. Fixations on developmental prosopagnosic trials were shifted more toward peripheral regions. When the classifier trained to discriminate acquired prosopagnosia from controls was asked to analyze the developmental prosopagnosic trials, the latter were classified as being more like control scanpaths.

**Conclusion:**

Only a few fixations during face scanning are required to differentiate controls from acquired prosopagnosia, with the latter showing anomalous biases. Developmental prosopagnosic scanpaths resemble degraded control scanpaths rather than anomalous biases. This study shows the potential of deep learning to identify abnormal behavioral markers in a disorder of complex visual processing.

## Introduction

When subjects look at scenes or objects, they often scan them with a series of fixations directed at different locations. These scanpaths reflect an active process in which saccades serve to direct both attention and the high spatial and color discrimination of the fovea to regions of greater interest (1). Many factors contribute to what makes a region ‘interesting’. This includes both bottom-up stimulus properties, whose distribution in a scene can be plotted in a salience map (2), as well as top-down cognitive processes such as memory (3), schematic knowledge (4, 5), and task-related processes (6). This complex interplay likely converges to maximize the rate of information accrual for perceptual decisions relevant to the current goals of the observer. This complexity also results in a high degree of variability in scanpaths, even for repeated views of the same image by the same viewer (7, 8).

The fact that a subject’s cognitive processes contribute to shaping scanpaths suggests that the latter may be a useful tool for exploring perceptual or cognitive dysfunction. Schematic knowledge refers to what the subject knows about the organization of certain type of scenes and where useful information is spatially distributed—for example, when looking for a cup, knowing that these are more often located on tabletops than on couches. Thus a subject’s fixations are guided more in a ‘top-down’ manner by a region’s relevance to a perceptual task than in a ‘bottom-up’ manner by its salience, which refers to properties or features that make it noticeable (9). Subjects with general visual agnosia may lose this schematic information, so that their scanpaths are guided more by salient cues than by scene knowledge (10).

Schematic knowledge is relevant to objects as well as scenes. With faces, studies show that the most informative regions for identifying people are the eyes, while the mouth region may be more useful for recognizing certain expressions (11–13). Accordingly, fixations are directed more to the eyes when healthy subjects process identity and more to the mouth when they switch to judging expressions in the same faces (6).

Subjects with prosopagnosia are impaired in recognizing the identity of faces. This can be an acquired or developmental problem (14, 15). There are functional subtypes, with apperceptive, associative and amnestic variants that differ in the degree to which the primary impairment lies in the perceptual processing of faces or in the access to facial memories (16). One intriguing question is whether any or all of these types of prosopagnosia are associated with degraded facial schematics. That is, do prosopagnosic subjects still possess a generic face representation that shows where useful information is likely to be located in a face? Face representations are often conceived of as situated in a multi-dimensional ‘face space’ (17), perhaps referenced to a normative ‘average face’ (18), with individual faces defined by their distances along those various facial dimensions. These distances likely determine which dimensions or facial properties are most useful in distinguishing individuals, generating a facial schematic for identity processing. If prosopagnosia results in a loss of this facial schema, this may be reflected in anomalous scanpaths during a face recognition task.

Some studies have indeed suggested that at least some prosopagnosic subjects have anomalous scanpaths when looking at faces (19–26). Most of these examined small numbers of subjects, though, sometimes single cases, which is problematic given that facial scanpaths vary considerably between healthy subjects (27, 28), and even differ for different faces scanned by the same subject (7, 29). Also, the prosopagnosic reports differ in the abnormalities they find, with some even noting normal scanning patterns (30, 31). The situation is complicated further by the fact that scanpaths have not just a spatial distribution, but also a temporal sequence, which is less often assessed—e.g. (19)—though it can reveal what regions are prioritized.

To advance on this situation, it would be desirable to assess larger numbers of prosopagnosic subjects making a large number of face scanning trials, and to use an analytic technique that can detect predicted and unanticipated differences in complex spatiotemporal sequences in large datasets with substantial between-trial and between-subject variability. Deep learning methods are one such analytic technique. Advances in deep learning algorithms have led to their recent deployment in clinical studies (32), including the fields of ophthalmology and neuro-ophthalmology, where they have been used to analyze ocular fundus images for disease (33–39, 40) and eye movements for cerebellar disorders (41). Most relevant to us, one study used a machine learning approach to explore the scanning of individuals with autism spectrum disorders while they viewed faces during an emotion recognition task, finding that these subjects fixated less on the eyes (42), while another used neural network models trained on scanpaths made by children looking at short videoclips to discriminate subjects with autism spectrum disorders (43).

Similar to that last study (43), we trained convolutional neural networks on images of scanpaths collected from healthy individuals and prosopagnosic subjects, but during a face recognition task. Our questions were first, whether the scanpath classifier could reliably discriminate prosopagnosic from control trials, and second, what scanpath properties the classifiers used to make that discrimination. One valuable feature of this study is that we used ocular motor data that had been previously evaluated with hypothesis-driven analyses (26, 44), allowing us to compare directly a deep-learning approach with traditional statistical methods. In particular, those prior studies tested the hypothesis that, compared to controls, prosopagnosic subjects would show more scanning of the lower than the upper face. However, the results showed little if any difference in the ratio of upper versus lower face scanning. One aim of this study was to explore whether a data-driven approach with deep learning might reveal differences either not evident or not hypothesized *a priori* in our prior reports.

Our study includes three sections. In the first section, we trained models to distinguish subjects with acquired prosopagnosia from healthy controls. The goals of this section were first, to determine the optimum number of fixations in a scanpath for classification performance; second, to discover the group features learned by the models; and third, to determine how the model labeled individual prosopagnosic subjects—that is, at a single-subject level. In the second section, we trained models to distinguish subjects with developmental prosopagnosia from healthy controls, with similar goals. Thus, these two sections generated two separate binary classifications, rather than a single three-class classification. By examining acquired and developmental prosopagnosia cohorts separately but with the same method, we asked whether these two variants showed similar anomalous scanning that would suggest degradation of facial schemata.

Finally, in the third section, we asked how the model that had been trained to distinguish acquired prosopagnosia from controls would classify subjects with developmental prosopagnosia. There is debate on whether developmental prosopagnosia represents a distinct pathologic entity or merely the low end of the normal spectrum of face recognition (15). If the scanpaths of developmental prosopagnosia resembled the anomalous scanning seen in acquired prosopagnosia, this might be more consistent with pathology disrupting facial representations in a similar way, despite the lack of visible structural damage. If they resembled those of healthy subjects more, this might fit more with the normative view.

## Materials and methods

### Subjects

Prosopagnosic patients were recruited through www.faceblind.org. The cohort with acquired prosopagnosia consisted of 8 subjects (3 female), four with occipitotemporal lesions (three bilateral), and four with anterior temporal lesions only (two bilateral). The group with developmental prosopagnosia had 10 subjects (7 female). As controls we had 20 age-matched healthy subjects (10 female). All subjects had corrected visual acuity of at least 20/30 in their best eye, with no history of psychiatric or other neurodegenerative conditions. All subjects were of white ancestry [prior studies have noted that east Asians and black subjects in England scan faces differently, with more emphasis on the center of the face (45, 46)].

Prosopagnosic subjects underwent a neuro-ophthalmological examination, Goldmann perimetry, and a battery of neuropsychological tests of intelligence, memory, attention, visual perception, and language skills (47), to exclude more general deficits that could account for face recognition difficulties. Subjects with developmental prosopagnosia also had to score less than 32 on the Autism Questionnaire (48), given that subjects with autism spectrum disorders can have impaired face recognition (49, 50), for reasons that may differ from developmental prosopagnosia.

Inclusion criteria for acquired prosopagnosia were performance worse than 2 standard deviations from the mean of control subjects on at least one of two tests of familiarity for recently viewed faces, the Cambridge Face Memory Test (51) or the face component of the Warrington Recognition Memory Test (52), while performing normally on the word component of the latter (Table 1).

**Table 1:** Demographic and clinical information for prosopagnosic subjects.

Subject	Age	Gender	Field	CFMT	WRMT			Lesion
					Faces	Words	Diff	
Acquired prosopagnosia								
R-IOT4	62	M	LUQ	**22**	46	50	4	Stroke
B-IOT2	60	M	BHH	**24**	**21**	42	**21**	Subdural hematoma
L-IOT2	59	M	Full	**21**	**27**	42	**15**	Left fusiform resection
B-ATOT2	23	F	Full	**24**	**19**	39	**20**	HSV Encephalitis
R-AT2	34	F	Full	**33**	**27**	47	**20**	HSV Encephalitis
R-AT3	37	M	Full	**31**	**31**	47	**16**	HSV Encephalitis
B-AT1	25	M	Full	**30**	**27**	45	**18**	HSV Encephalitis
B-AT2	47	F	Full	**31**	**31**	46	**15**	Trauma, temporal resection
Developmental prosopagnosia								
DP014	42	M	Full	**32**	30	48	**18**	
DP016	52	F	Full	**41**	37	49	**12**	
DP021	29	F	Full	**37**	33	50	**17**	
DP024	35	F	Full	**41**	38	50	**12**	
DP033	46	F	Full	**29**	39	50	**11**	
DP035	40	M	Full	**32**	35	49	**14**	
DP038	27	F	Full	**39**	36	49	**13**	
DP044	36	F	Full	**40**	34	49	**15**	
DP039	50	M	Full	**22**	46	50	**4**	
DP202	20	F	Full	**33**	**32**	50	**18**	

LUQ, Left Upper Quadrant; BHH, Bilateral Homonymous Hemianopia; HSV, herpes simplex virus; CFMT, Cambridge Face Memory Test; WRMT, Warrington Recognition Memory Test; diff, subtraction of face from word score on the WRMT; BHH, bilateral hemifield defect; LUQ, left upper quadrantanopia; HSV, Herpes simplex virus; Bold, abnormal score.

Subjects with developmental prosopagnosia reported life-long difficulty in face recognition, corroborated by a high score on the 20-item Prosopagnosia Index (53). This was supplemented by objective confirmation of impaired face recognition with at least two of the following: (i) a discordance between preserved word memory and impaired face memory on the Warrington Recognition Memory Test that was in the bottom 5th percentile, (ii) a score at least two standard deviations below the control mean on the Cambridge Face Memory Task, (iii) a score at least two standard deviations below the control mean on an Old/New faces test (54) or (iv) a score at least two standard deviations below the control mean on a Famous Faces Test.

The demographic details of our acquired and developmental prosopagnosic subjects have been reported previously (26) and are summarized in Table 1. The location of lesions in the acquired prosopagnosic subjects is indicated by their identifiers. ‘*R’* indicates a right-sided lesion, ‘*L’* a left sided lesion, and ‘*B’* bilateral lesions, while ‘*IOT’* indicates an inferior occipitotemporal lesion, ‘*AT’* an anterior temporal lesion, and ‘*ATOT’* a combination of the two.

### Data acquisition

An Eyelink 1,000 (SR Research Ltd., Mississauga, Canada) tracked the eye movements of subjects while their head was stabilized by a chin rest in a dimly lit room. Subjects were placed 34 cm away from a computer screen with a 1,024 × 768 pixel resolution, and refresh rate of 140 Hz.

### Protocol

The research ethics boards at the University of British Columbia and Vancouver General Hospital approved the research protocols and all subjects gave written consent in accordance with the Declaration of Helsinki. As this work involves data collected several years ago (26), no part of the study procedures or analyses had been pre-registered prior to the research being conducted.

In the learning phase subjects saw 10 facial images, of five people each with two expressions. This was followed by a recognition phase with 35 trials, 10 showing the learned faces and 25 showing distractor faces of different people. All learning and recognition trials began with the subject fixating a cross 7° above where the face would appear. In both learning and recognition phases, subjects were allowed to scan the faces for as long as they wished before pressing the spacebar to move to the next trial. For analysis we collected fixations from the period beginning with the appearance of the face stimulus and ending with the pressing of the spacebar, resulting in a wide range in the number of fixations across trials. Since deep learning methods benefit from larger sample sizes, we entered scanning data from both the learning phase and the recognition phase into our models. This was justified because the prior study had shown that there were no main effects of phase or group-phase interactions in the distribution of fixations, as measured by upper/lower, eye/mouth, or central/peripheral indices (26).

### Stimuli

Faces were taken from the KDEF Face Database (76).^1^ For the five male target people whose faces were being memorized, we chose images with neutral, sad, or happy expressions. For the distractor faces, 25 male facial identities were chosen at random, also with varying expressions. All stimuli were presented on a white background, with the tip of the nose centered on the screen.

### Data preprocessing

The X and Y coordinates and the duration of each fixation were recorded. Each fixation was classified as being directed to one of 10 facial regions of interest, namely the left or right eyes, eyebrows, or cheeks, as well as the forehead, nose, mouth or chin. We imported the data into a Python environment, where trials were stored in individual Pandas Data Frames. For each trial, fixations were considered as separate dimensions, resulting in a vector whose dimensions were equal to the number of fixations in that trial. Each dimension stored a categorical value between 1 and 10 for the facial region to which the fixation was directed.

### Machine learning methods

#### Section 1: deep learning classification of acquired prosopagnosia vs. controls

We first designed a *Baseline Model* to classify acquired prosopagnosic and control trials based on scanpaths. The aim was to represent each trial by the spatiotemporal characteristics of the fixations made by the subject. Importantly, this did not include all the fixations made in a trial, but only the first *m* fixations. We transformed each trial to an *n-*dimensional vector. The first *m* dimensions represented the trial’s first *m* fixations as numerical variables indicating to which of the 10 facial regions each fixation had been directed. Dimensions *m + 1* through *n-1* represent engineered features (Appendix). The last dimension *n* represented the dependent variable, which was subject group (acquired prosopagnosia vs. control).

To determine the optimal number of fixations to use for image classification, we systematically varied the number of fixations *m* included for each trial, where *1 < m ≤ 20*, to train a logistic regression classifier (the *Baseline Model*). This allowed us to examine the area under the curve (AUC) for discriminating prosopagnosic from control trials as a function of the number of fixations used.

**Figure 1:**
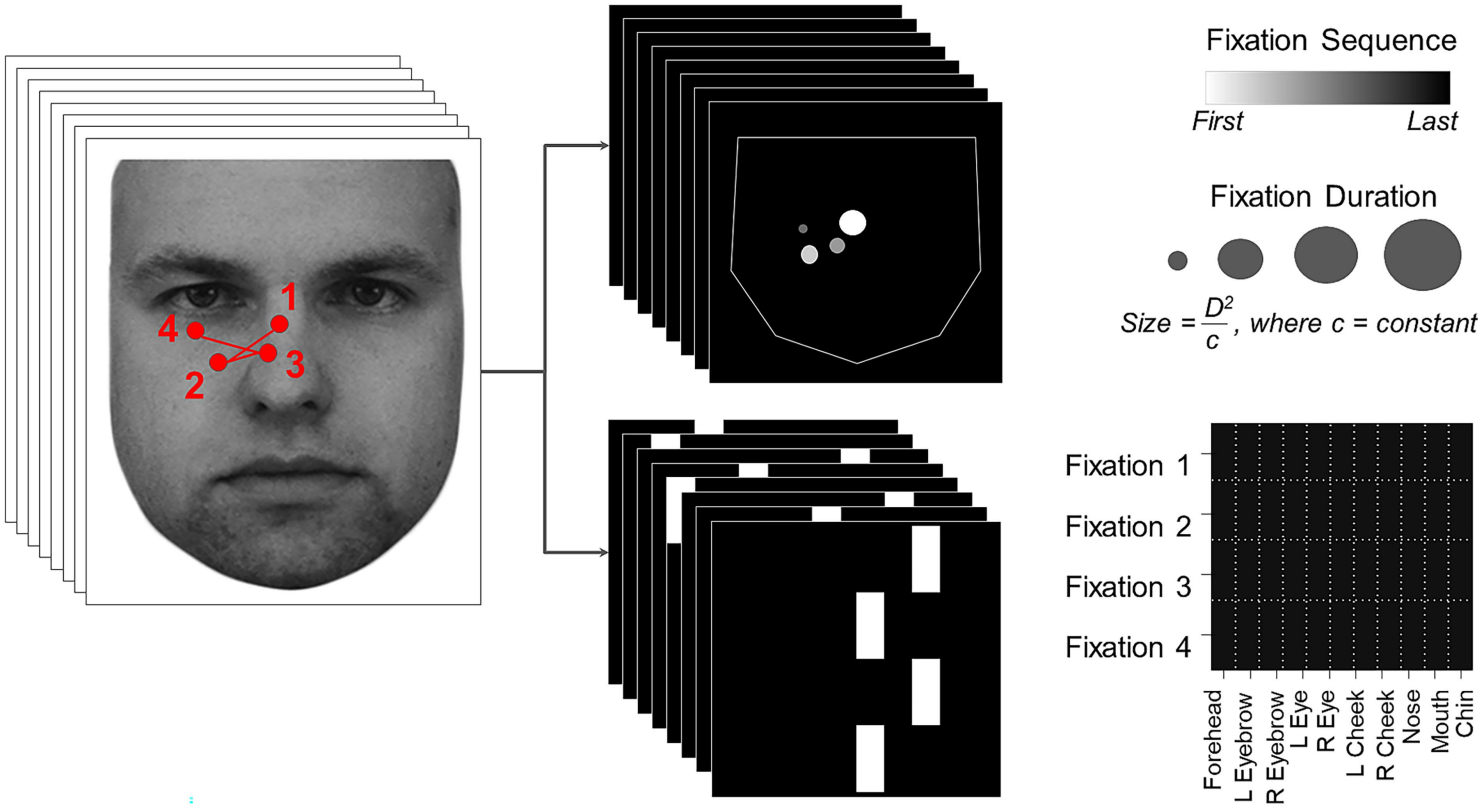
On the left, an example scanpath is shown overlaid on the face (KDEF image ID AM02NES) being viewed. This is transformed into two image representations on the right, the *Facial Scanpath* (top) and the *ROI Sequence* (bottom). Reproduced with permission from Lundqvist et al. (76).

Scanpaths have both spatial and temporal aspects, which can be represented in a single static image in a number of ways. These will differ in the information they emphasize. Since the best means of discriminating prosopagnosic from control scanpaths was not known *a priori*, we generated two types of image representations of the scanpath of each trial (Figure 1), using the optimal number of fixations for classifying acquired prosopagnosic subjects versus controls that had been identified by the *Baseline Model*.

*Facial Scanpath* (Figure 1, *top*): This plotted fixations as discs centered on the original X and Y coordinates of each fixation. The radius of each disc was proportional to the square of the duration of fixation (to amplify duration differences) divided by a constant (to ensure that most discs still lay within the face boundaries). The order of fixations in the sequence was indicated by the brightness of the disc, with earlier fixations being brighter. This method thus represents both fixation order and duration, and shows the spatial location of fixations as a continuous variable.

*ROI sequence* (*Figure 1*, *bottom*): The x-axis showed the facial region of interest to which the fixation was directed, while the y axis showed the fixation’s position in the temporal sequence, with the first fixation at the top and the last at the bottom. This type of representation emphasizes the temporal order more strongly than the *Facial Scanpath* does, and represents location categorically by feature rather than continuously, while omitting fixation duration.

We then trained two deep convolutional neural network models using each of these two image sets. The model architecture was identical in all aspects (besides the input size) for both. Our convolutional neural networks used filters that had a 3×3 kernel with a stride length of 2, and a 3 × 3 maxpool size. There were three convolution layers, the first using 32 feature maps, the second 64, and the last 128. The learning rate was set at 0.0001. Code for this analyses is available as a GitHub repository for this project: https://github.com/akazemian/Scanning-Faces-and-Prosopagnosia

The training step for each model involved an 8-fold cross-validation procedure to ensure that the model was tested on the trials from each of the 8 prosopagnosic subjects once. The k-fold validation technique splits the data into k-subsets and training/testing is repeated k times, where each of the k subsets is used once as test set and the other k-1 subsets are used for the training. In our study, a subset is the set of data from one prosopagnosic subject. For the acquired prosopagnosia section, there are 8 folds because there are 8 subjects. Each time, we train on 6 subjects, validate on 1 of the omitted subjects and test on the other omitted subject. We repeat until all subjects have been used once for validation and once for test. For each fold, the process was repeated 10 times, each time with a new random order of trials and different randomly initialized weights in the learning phase, to assess the reproducibility of the results, and the performance of the model was averaged across the 10 repetitions in each fold. Since the dataset involved only 2 groups, a binary loss function was used in the model architecture, measuring the performance as a probability value. The cross-entropy loss increased as the predicted probability diverged from the actual probability, and the function was calculated as


−1n∑i=1nyilogpi+(1−yi)log(1−pi)

where *n* is the number of scalar values in the model output and $y_{i}$ and $p_{i}$ are the *ith* actual value and predicted value in the model output, respectively. The more confident the model is about a trial’s label, the closer the predicted value is to 0 (Control) or 1 (Prosopagnosic).

Having trained two models on the two sets of images (*Facial Scanpath and ROI Sequence*) for each fold, we next used a weighted average of the predicted values from each model to maximize classification performance for each fold. This we called the *Hybrid* model. The optimal weights for each of the two models were found using a non-negative coefficient linear regression model. In each cross-validation fold, the linear regression model was trained on the predicted values from the two models and evaluated using the true labels. The positive coefficients were enforced to disregard inverse correlations between the predictions and the true labels. The linear regression coefficients that resulted in the highest AUC score were selected as the optimal weights of the 2 models. In this way, the weights of the two models varied in each fold based on the models’ performance in that fold.

#### Section 2: deep learning classification of developmental prosopagnosia vs. controls

The same procedure as in Section 1 was used, now with developmental prosopagnosia versus controls. We started by using a *Baseline Model* with the same vector dimensions to find the optimal number of fixations for classification, and used this optimal number to generate the same two types of scanpath images. We then trained two convolutional neural networks and developed the *Hybrid* model. One difference from Section 1 is that there were 10 developmental prosopagnosic subjects, whereas there were only 8 acquired prosopagnosic subjects. This led to a 10-fold cross-validation design for training the convolutional neural networks.

#### Section 3: classification of the scanpaths in developmental prosopagnosia by the hybrid model trained to distinguish acquired prosopagnosia from control subjects

Here the *Hybrid* model from section one was asked to classify the trials from developmental prosopagnosia subjects as being either more like acquired prosopagnosia trials or more like control trials. The two image representations of a trial were fed to all eight *Facial Scanpath* and *ROI Sequence* models, and the final prediction was calculated as the average of the 8 outputs. The more similar a developmental prosopagnosic trial was to a control trial, the closer the final prediction was to 0, and the more similar it was to an acquired prosopagnosic trial, the closer the prediction was to 1.

### Additional statistical analysis

To confirm the statistical significance of the results obtained from our models, we used a permutation test to compare the *Hybrid* model’s AUC score with chance performance in sections one and two. This was done by training the *Facial Scanpath* and *ROI Sequence* models on shuffled labels, followed by testing them on true labels and recording the AUC using the same cross-validation design. This process was repeated 100 times to obtain a distribution of chance AUC scores. A *p*-value was then determined based on the number of permutations whose score was better than the true *Hybrid* AUC.

We also performed two *post-hoc* analyses in section one to confirm the results depicted by the outputs of the convolutional neural networks. For Section 1, the analysis for acquired prosopagnosia, we constructed left: right eye indices for each subject as before (26, 44), by subtracting the number of fixations to the eye on the right side of the image from the number to the eye on the left, and dividing by the sum of the two. A positive index indicates more fixations on the eye on the left side, which is the photographed person’s right eye. We analyzed this index with a one-tailed t-test for the *post-hoc* hypothesis that prosopagnosic subjects fixate the eye on the right of the image more than controls do. Second, we constructed and analyzed an eye: mouth index, where a positive index indicates more fixation on the eyes, and subjected this to a one-tailed t-test for the *post-hoc* hypothesis that acquired prosopagnosic subjects scan the mouth more than the eyes, compared to controls.

In Section 2, the analysis for developmental prosopagnosia, we constructed a dispersion index (26). This measured the distance of each fixation from the center of the face, and calculated the standard deviation of these distances for each trial, finally obtaining an average of this measure of variability across all the trials for each subject. A large average standard deviation indicates that subjects are scattering fixations broadly, while a low one indicates that they are fixating a few areas repeatedly. We analyzed this dispersion index with a one-tailed t-test for the *post-hoc* hypothesis that developmental prosopagnosic subjects had more dispersed scanning.

## Results

### Behavioral results

Mean discrimination performance (d’) was 1.82 (s.d. 0.99) for controls, 1.63 (s.d. 0.76) for developmental prosopagnosia, and 0.52 (s.d. 0.63) for acquired prosopagnosia (Table 2; Supplementary Figure 1). An ANOVA showed a main effect of group [*F*_(2, 35)_ = 6.58, *p* < 0.004]. The acquired propagnosia group performed worse than either the controls (*p* < 0.003) or the developmental prosopagnosia group (*p* < 0.034), while there was no difference between the last two. As noted (26), this short-term familiarity protocol is easier than other tests of face recognition, and was not designed to maximize diagnostic accuracy for prosopagnosia. Nevertheless, the fact that subjects with acquired prosopagnosia performed worse than those with the developmental variant is consistent with our prior observations with other tests (55, 56).

**Table 2:** Recognition performance of subjects during the task, d’ being discriminative sensitivity, and c being criterion bias.

		d’	c
Prosopagnosia acquired			
RIOT4		0.47	−0.23
BIOT2		0.47	0.29
LIOT2		−0.69	−0.50
BATOT2		1.33	0.62
RAT2		0.51	0.00
RAT3		0.71	−0.35
BAT1		0.14	0.77
BAT2		1.23	−0.09
Developmental			
DP014		1.03	0.77
DP016		0.92	−0.71
DP021		2.69	−0.06
DP024		1.31	0.19
DP033		2.12	0.22
DP035		1.09	0.29
DP038		2.35	0.47
DP044		0.40	0.05
DP039		2.28	0.14
DP202		2.05	−1.03
Controls	Mean	1.83	0.04
	(s.d.)	0.98	0.45
C1		3.40	−0.05
C2		3.40	−0.05
C3		3.03	−0.23
C4		2.82	0.23
C5		2.59	−0.45
C6		2.46	0.05
C7		2.28	−0.61
C8		2.11	0.59
C9		2.11	0.59
C10		1.86	0.35
C11		1.84	−0.08
C12		1.70	−0.33
C13		1.68	0.00
C14		1.39	0.95
C15		1.03	0.77
C16		0.99	−0.50
C17		0.99	0.35
C18		0.78	0.14
C19		0.18	−0.62
C20		−0.10	−0.20

#### Section 1: deep learning classification of acquired prosopagnosia vs. controls

We trained our *Baseline Model* on a dataset generated using the first *m* fixations of all trials, for *1 < m ≤ 20* and plotted AUC as a function of *m*. We expected two factors to contribute to the shape of the obtained graph. First, with more fixations per trial, the amount of scanpath information increases, which could improve the ability of the model to distinguish between the two subject groups. On the other hand, since we discard trials with fewer than *m* fixations, a higher *m* means a smaller dataset, which could cause a drop in classification performance. Hence, we expected an inverted-U shape to the AUC function, with classification performance peaking at a certain number of fixations. This was indeed the case, with the first four fixations being the optimal number.

**Figure 2:**
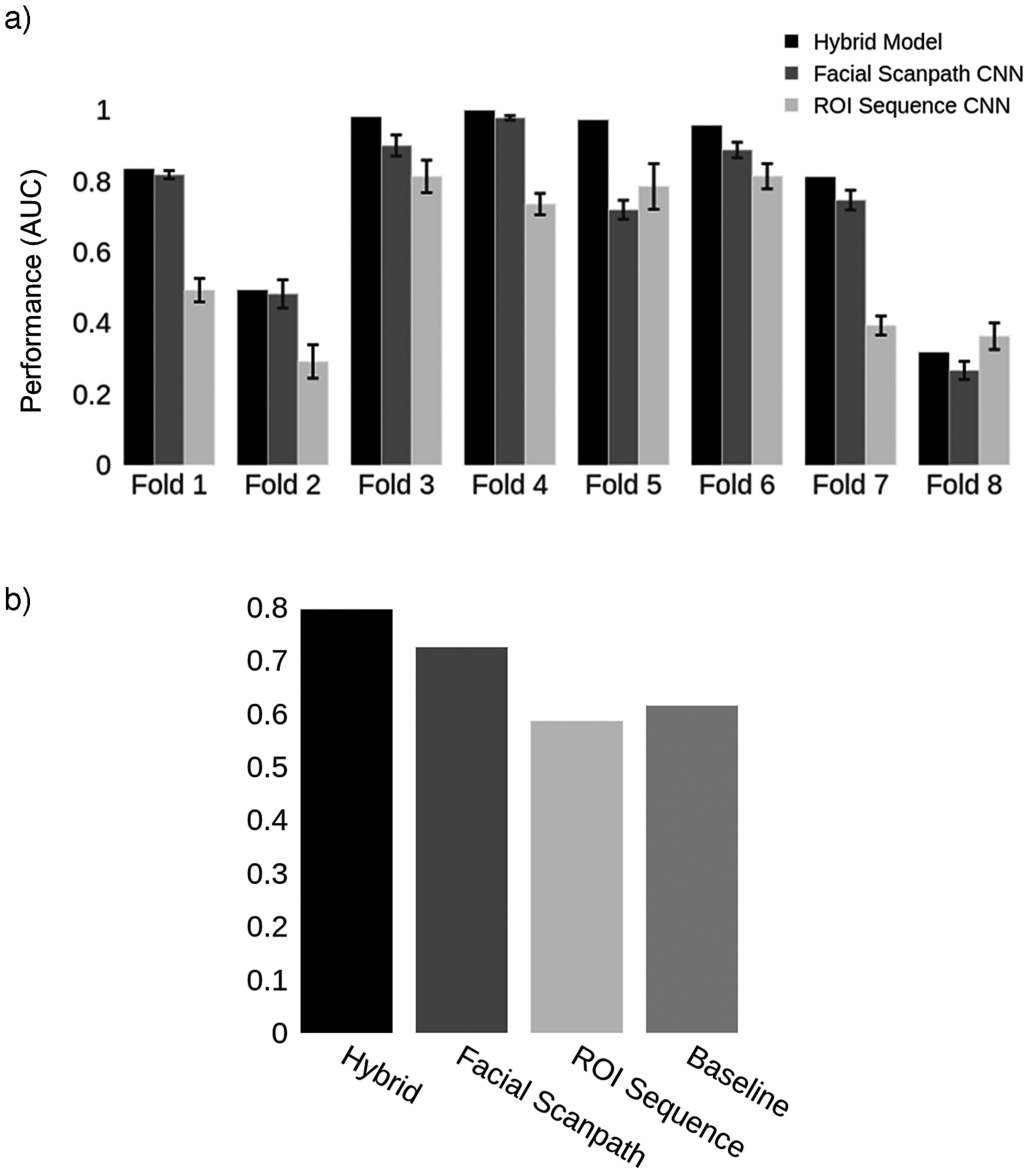
Acquired prosopagnosia. **(a)** Classification results for the two models in each fold, as well as the *Hybrid* model. Error bars represent one standard deviation from the mean score obtained by repeating the train-test process 10 times for each fold, evaluating the reproducibility of the results. The *Hybrid* model was generated using the average predictions from the two models in each fold. **(b)** Mean AUC score for models in **(a)** and the *Baseline* using the first 4 fixations.

We trained two convolutional neural networks, one using the *Facial Scanpath* and the other the *ROI Sequence* image sets. The *Facial Scanpath* model performed better than the *ROI Sequence* model, with one of the folds even achieving an almost perfect AUC score of 1 (Figure 2A). The *Hybrid* model’s AUC was recorded and averaged across the 8 folds, with an overall AUC of 80% (Figure 2B).

**Figure 3:**
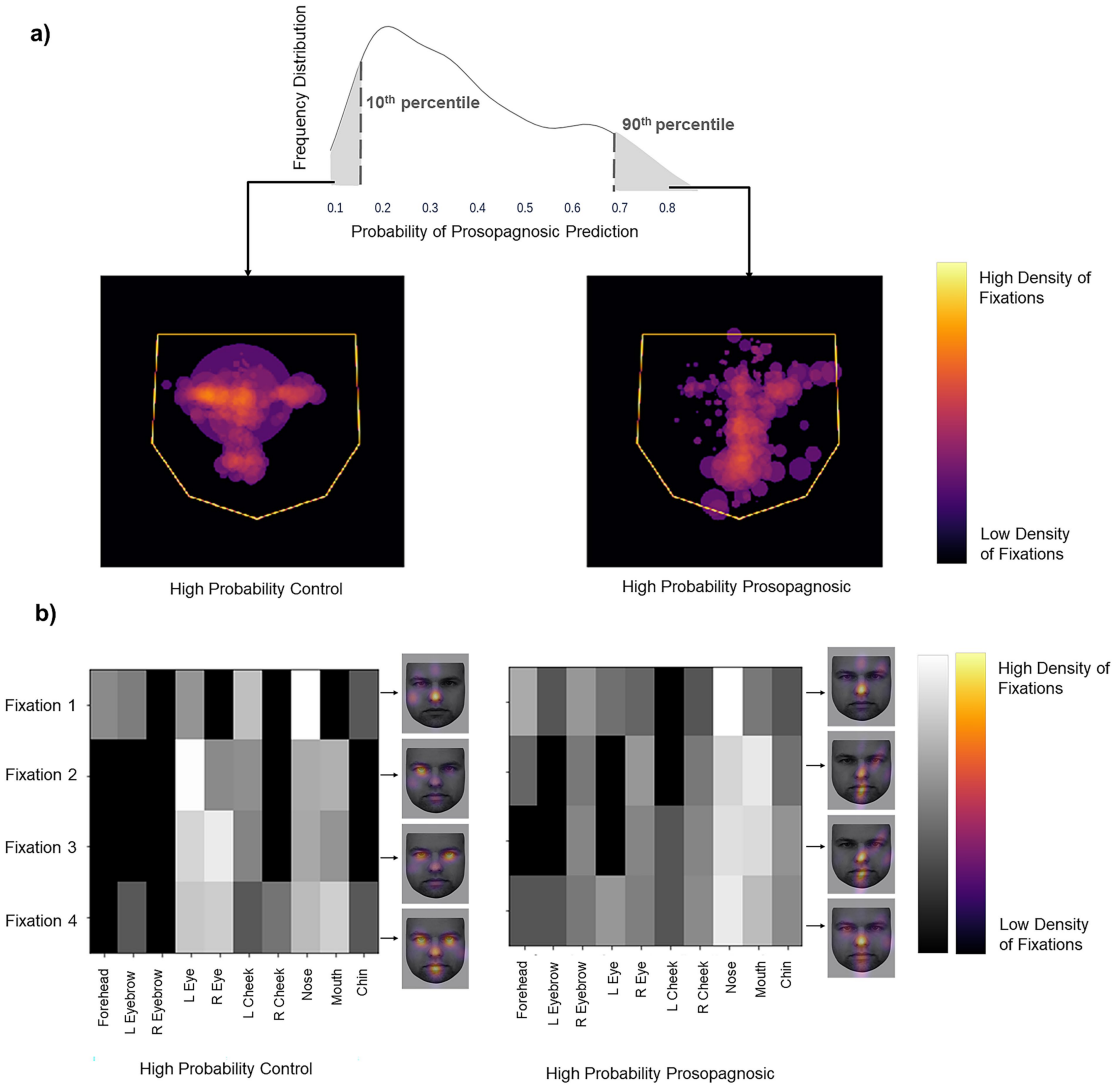
**(a)** Left image shows the superimposition of all trials that the *Facial Scanpath* model classifies as control (i.e., label 0) with high confidence (<10th percentile of all predicted values), while the right shows that of trials that the model classifies as acquired prosopagnosic (i.e., label 1) with high confidence (>90th percentile of all predicted values). **(b)** Superimposition of all trials that the *ROI Sequence* model classifies as control (label 0) with high confidence and trials that it classifies as prosopagnosic (label 1) with high confidence. The brighter a cell, the higher the number of trials with a fixation on a particular ROI for that position in the fixation sequence. The faces next to each plot (KDEF Image ID AM02NES) illustrate this by showing the relative density in space for each of fixations 1–4, overlaid on a sample face.

To discover the features learned by the models we examined the trials that the models classified with high confidence. Each trial’s label was predicted as a probability. The more confident the model’s prediction, the closer the probability is to either 0 (control) or 1 (prosopagnosia). We looked at the trials with predictions either below the 10th percentile (high confidence for control) or above the 90th percentile (high confidence for prosopagnosia) of each fold. We overlaid these trials (obtained from all subjects regardless of their group membership) to see what model thought constituted a prosopagnosic and a control scanpath. The *Facial Scanpath* model showed that control-label scanpaths had more fixations on the eyes, particularly the eye on the left side of the image, while prosopagnosic-label scanpaths fixated the nose, mouth and right upper face more (Figure 3A). The *ROI Sequence* model showed that both groups fixated the nose first, then control-label scanpaths shifted to the left and then both eyes, while prosopagnosia-label scanpaths stayed on the nose and mouth more (Figure 3B).

**Figure 4:**
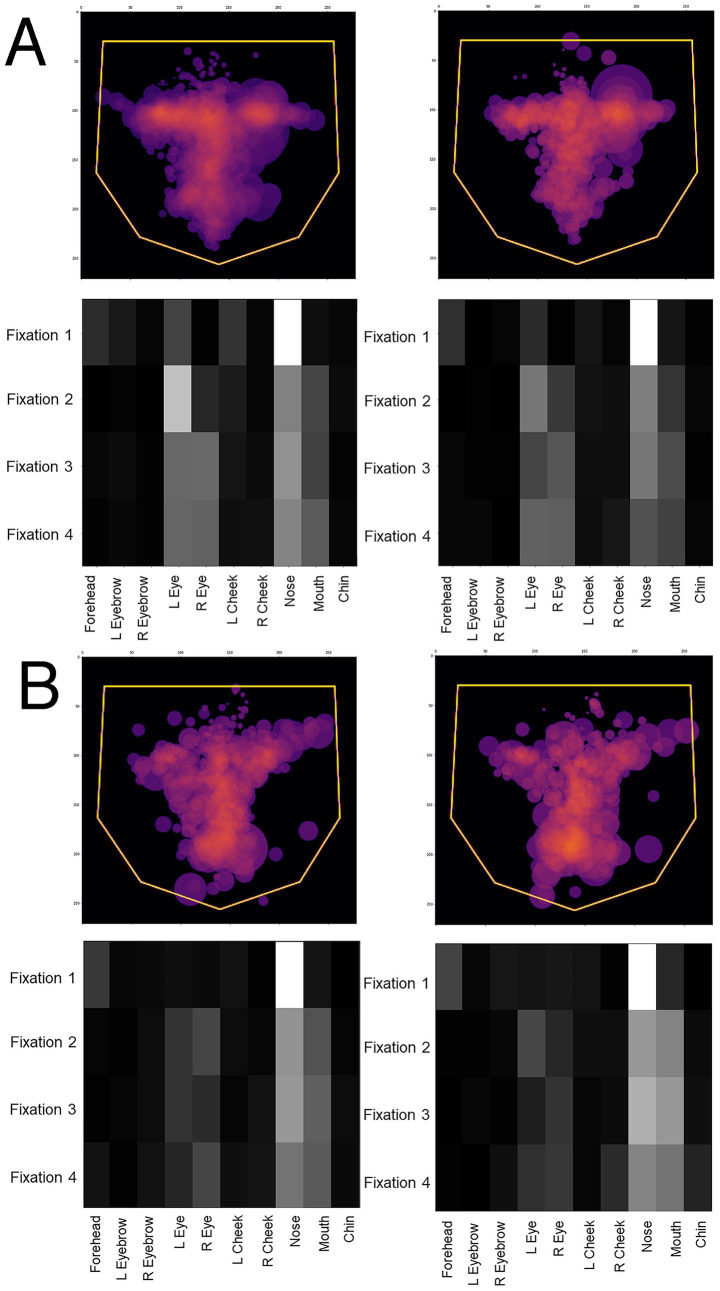
Overlay images of the scanpaths of the first four fixations of **(A)** control subjects and **(B)** subjects with acquired prosopagnosia, during correct trials (left graphs) and error trials (right graphs). For **(A,B)**, the top shows the *Facial Scanpath* overlay, the bottom shows that of the *ROI Sequence*. Scanpaths during correct and error trials of subjects with acquired prosopagnosia resemble each other more than they do the scanpaths of controls on error trials, which again do not appear much different than how control subjects scan faces on correct trials.

One potential confound is that, since prosopagnosic subjects make more errors than controls, could the model’s predictions actually be discriminating between correct and incorrect trials, rather than between control and prosopagnosic trials? To address this we looked at the scanning on correct and incorrect trials separately. If the model was actually discriminating trial accuracy instead of group membership, scanning on error trials would differ from scanning on correct trials, rather than differing between prosopagnosic and control subjects. This was not the case (Figure 4). Rather, the scanning of controls was similar regardless of whether they gave the right or wrong answer, and the same was true for prosopagnosic subjects.

To assess the significance of the *Hybrid* model’s performance, we used a permutation test to compare the *Hybrid* AUC score with chance performance. Our model’s performance of 80% was significantly better than the performance of 100 chance models (*p < 0.01*): i.e., none of the models trained on shuffled labels performed better than the *Hybrid* model.

As a *post-hoc* application of traditional statistics for comparison, we analyzed how subjects distributed fixations between the right and left eyes, and between the eyes and mouth, limited to the first four fixations of each trial. (This differs from the prior study (26), which used all fixations of a trial.) The mean left: right index was −0.18 (s.d. 0.64) for subjects with acquired prosopagnosia, and 0.28 (s.d. 0.48) for controls, confirming that subjects with acquired prosopagnosia fixated the eye on the right side of the image more (*p* = 0.027). The mean eye: mouth index was 0.10 (s.d. 0.63) for subjects with acquired prosopagnosia, and 0.46 (s.d. 0.40) for control subjects, indicating a bias to the eyes for controls but more even scanning between the eyes and mouth in prosopagnosic subjects (*p* = 0.042).

**Figure 5:**
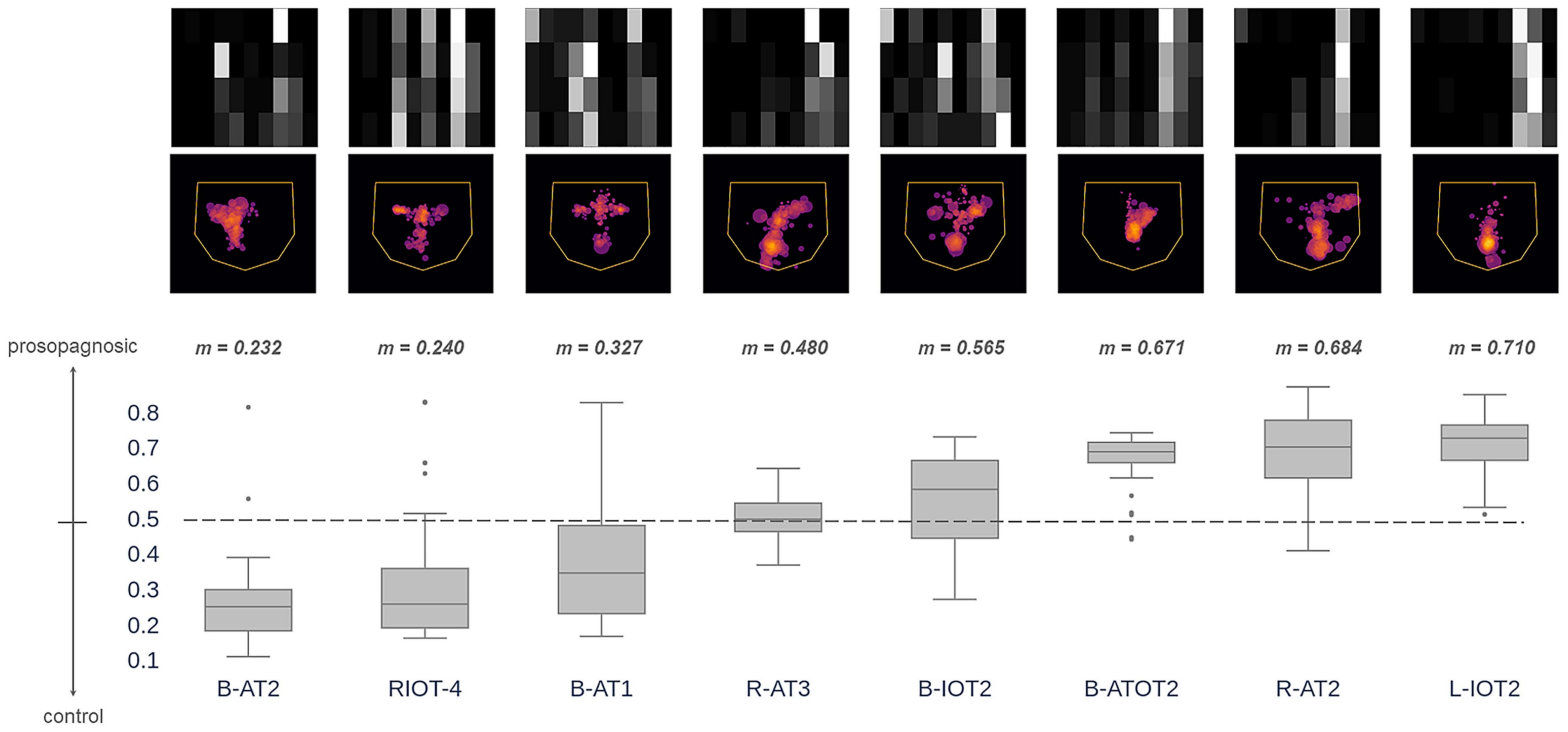
These images superimpose the first 4 fixations from all trials for each acquired prosopagnosic subject. The box plots represent the distribution of trial predictions from the *Hybrid* model for each subject. The ends of the box represent the lower and upper quartiles, while the median is marked by a line inside the box and the whiskers represent the minimum and maximum values, with outliers beyond these plotted as dots. Subjects are sorted by median prediction, which is the value *m* written at the top of each box. Thus, subjects on the left have fixation patterns that on average are closer to the model’s understanding of how a control subject fixates faces, while those to the right have characteristics that match the model’s understanding of prosopagnosic scanning behavior.

How did the *Hybrid* model classify individual prosopagnosic subjects? We examined the distribution of outputs for all eligible trials (those with 4 or more fixations) made by each individual subject (Figure 5). For any given subject, a median prediction closer to 1 than to 0 would indicate that most of the trials of that subject were similar to the model’s understanding of a prosopagnosic scanpath. There was substantial variation across subjects, with the scanpaths of a few (RIOT-4, B-AT1, B-AT2) appearing on average more like control scanpaths. Two points are worth mentioning. The likelihood that a subject had more prosopagnosic-like or more control-like scanpaths was neither related to the presence of a field defect (Table 1) nor to whether they had anterior temporal or occipitotemporal lesions.

#### Section 2: deep learning classification of developmental prosopagnosia vs. controls

**Figure 6:**
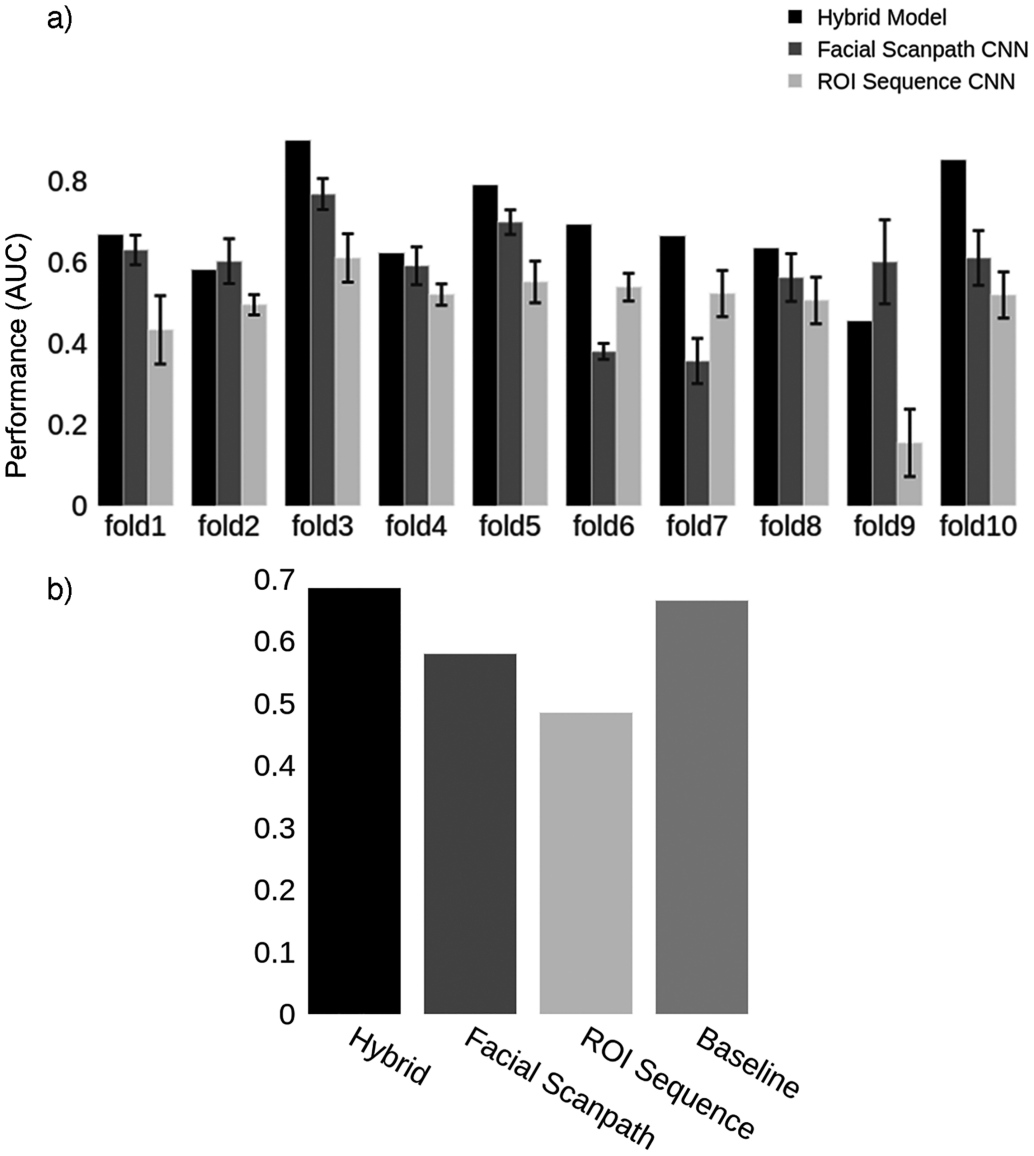
Developmental prosopagnosia. **(a)** Classification results for the two models in each fold, as well as the *Hybrid* model. Error bars represent one standard deviation from the mean score obtained by repeating the train-test process 10 times for each fold, evaluating the reproducibility of the results. The *Hybrid* model was generated using the average predictions from the two models in each fold. **(b)** Mean AUC score for models in **(a)** and the *Baseline* using the first 16 fixations.

In this section, we performed a similar process for the developmental prosopagnosia cohort. We started by finding the optimal number of fixations to use, which was 16. Next, we generated the same two types of image representations for developmental prosopagnosic subjects and controls using each trial’s first 16 fixations. This resulted in 16 fixation discs for each *Facial Scanpath* image, and a 16×10 matrix for each *ROI Sequence* image. Figure 6 shows the classification results for the 10 folds, including the *Hybrid* model, which achieved an average AUC of 69% (Figure 6B).

**Figure 7:**
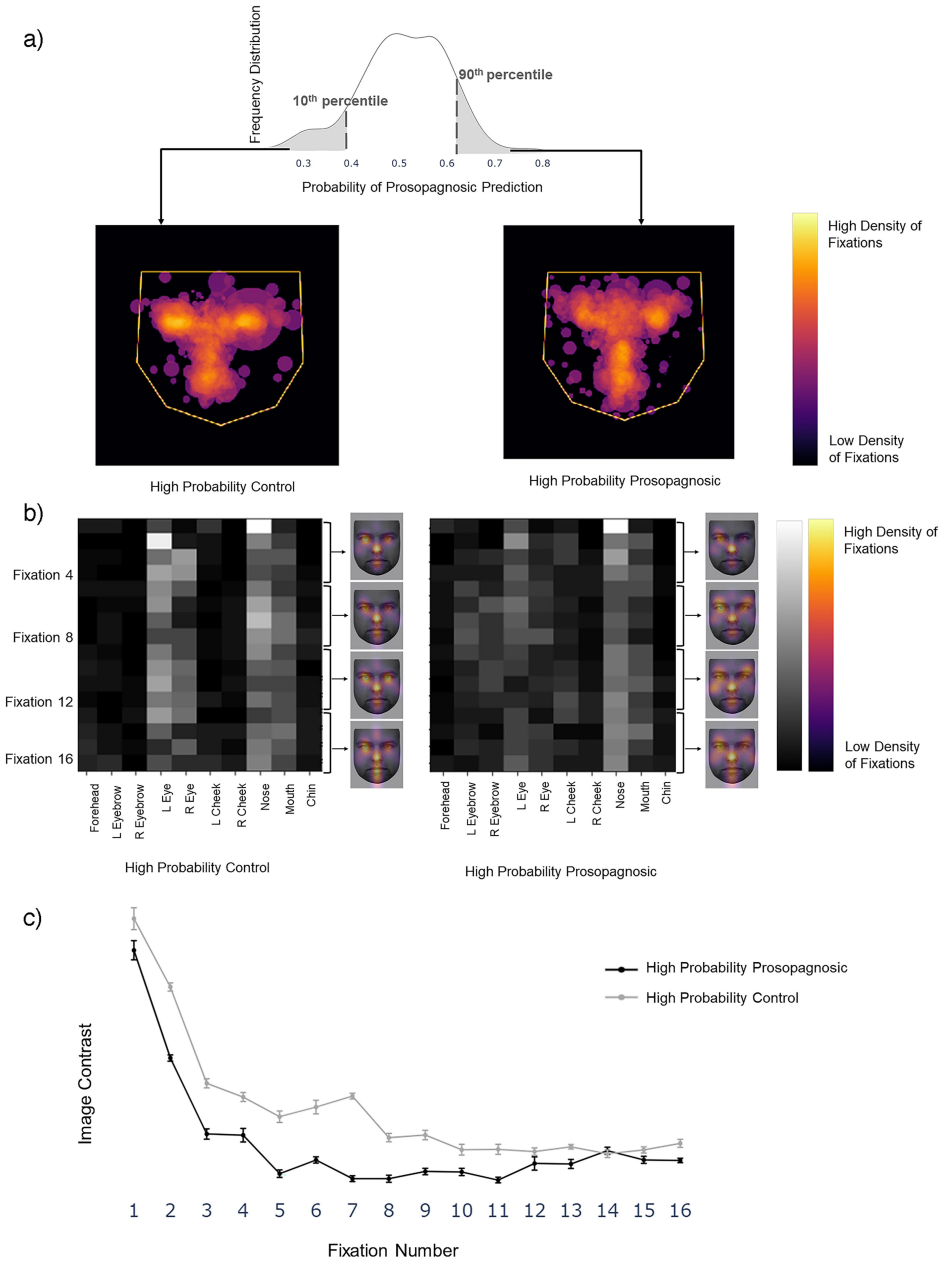
**(a)** Left image shows the superimposition of all trials that the *Facial Scanpath* model classifies as developmental prosopagnosic (i.e., label 1) with high confidence (>90th percentile of all predicted values), middle shows all trials labeled as Control (i.e., label 0) with high confidence (<10th percentile of all predicted values). **(b)** Superimposition for the *ROI Sequence* model, with conventions similar to Figure 3B. The face (KEDF Image ID AM02NES) heat maps here represent the average fixation density for three consecutive fixations at a time. **(c)** The line plot represents the mean contrast value for each fixation number in the 10 train-test repetitions, with the error bars representing 1 standard error.

Next, we visualized the scanpaths in the same way as in Section 1. The *Facial Scanpath* Model confidently classified trials with a higher density of fixations on the central features as control trials, and those with more peripherally scattered fixations as developmental prosopagnosia trials (Figure 7A). The *ROI Sequence* model suggested that the two groups have a similar scanning behavior during the initial fixations, with a high density of fixations on the nose followed by fixations on the left eye. This model classified trials with a higher concentration on the central regions and almost no fixations on the peripheral regions as control trials, and those with more evenly distributed fixations as developmental prosopagnosia trials (Figure 7B).

For a *post-hoc* statistical confirmation of this impression, we analyzed the contrast between the 10 ROIs for each fixation row in the sequence from 1 to 16 (Figure 7c). A high contrast value indicates a tendency to fixate some regions more than others, while a low contrast value indicates that all regions are fixated to a similar degree. Contrast decreased as the fixation sequence progressed, meaning that fixations became more widely distributed across the face, but that at most points in the fixation sequence the prosopagnosic-label trials had more evenly distributed fixations than the control-label trials. A two-way repeated-measures ANOVA with contrast as the dependent variable and group (prosopagnosic-like versus control-like) and fixation number as independent variables showed a significant main effect for group (*F* = 22.8, *p* = 0.001) and fixation number (*F* = 95.0, *p* < 0.0001). There was a significant interaction between the two (*F* = 2.57, *p* = 0.0021), due to a greater difference between groups for earlier fixations.

For a *post-hoc* application of traditional statistics for comparison, we analyzed the dispersion index. This differs from the prior study (26), which used all fixations of a trial, by including only the first 16 fixations of each trial, and only trials with at least 16 fixations. The mean dispersion index was 35.3 (s.d. 4.87) for subjects with developmental prosopagnosia, and 31.8 (s.d. 4.64) for control subjects, confirming that subjects with developmental prosopagnosia had more dispersed scanning [t_(24)_ = 1.82, *p* < 0.041].

**Figure 8:**
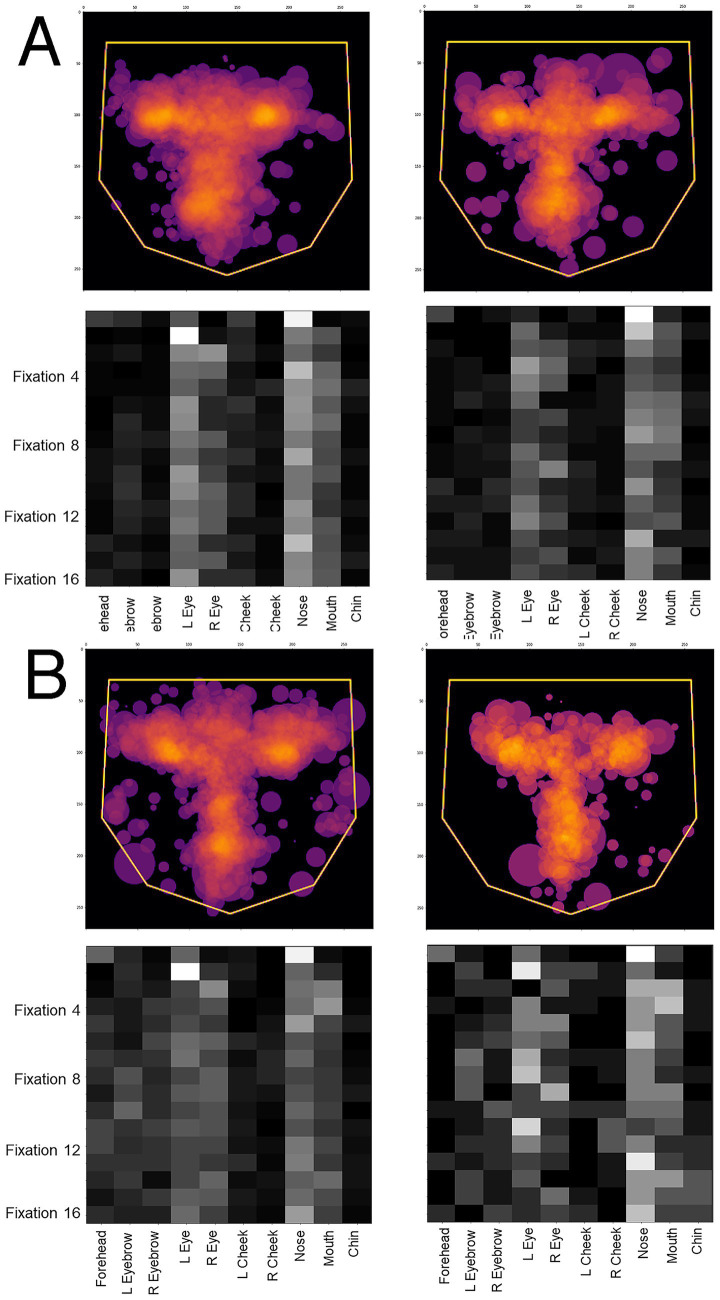
Overlay images of the scanpaths containing the first 16 fixations of **(A)** control subjects and **(B)** subjects with acquired prosopagnosia, during correct trials (left graphs) and error trials (right graphs). Top graphs of each half shows the *Facial Scanpath* overlay, bottom graphs shows that of the *ROI Sequence*.

As for acquired prosopagnosia, we compared the predictions to the overlay images for correct and incorrect trials. Again, the difference in the dispersal of scanning tracked more with the subject group than the accuracy of the trial response. The greater dispersal of scanning in developmental prosopagnosia is particularly evident in the *ROI sequence* plots (Figure 8).

#### Section 3: classification of developmental prosopagnosic scanpaths by the acquired prosopagnosia-control hybrid model

**Figure 9:**
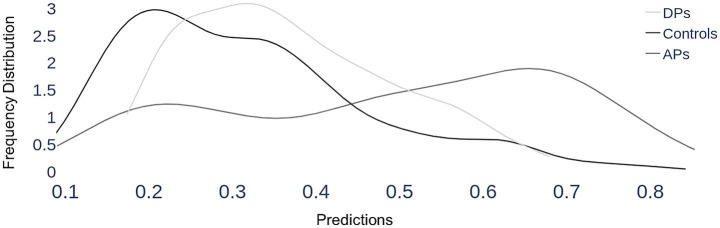
Section 3, group results. Each trial is classified by the *Hybrid* model (that was trained to discriminate between acquired prosopagnosia and controls) as having a probability of being a prosopagnosic-like scanpath, which runs from 0 (definitely control-like) to 1 (definitely prosopagnosic-like), which is represented on the x-axis. For each of the three groups, all the trials of all member subjects are included in a group plot, showing how these predicted probabilities are distributed for that group, depicted as a density line—i.e. the frequency of a particular predicted probability. As expected, the scanpaths of the control group have a mean probability of 0.31 and a peak close to the lower end, around 0.2, indicating that most of their trials show control-like properties. Acquired prosopagnosic subjects show a mean probability of 0.49, with a shallower peak around 0.66, indicating more tendency for their trials to be classified as prosopagnosic-like. The key finding is that developmental prosopagnosic trials have a distribution that appears in shape and place more similar to that of controls, with a mean probability of 0.37.

We asked how the *Hybrid* model that was trained to distinguish between acquired prosopagnosia and control subjects would classify the data of the developmental prosopagnosia group. We generated images using the first 4 fixations of each trial of the developmental prosopagnosic subjects and used them as input to the trained *Hybrid* model developed in Section 1. We first performed a group analysis. Again, the key output of the classifier is the predicted probability of group membership of a trial, ranging from 0 (control-like) to 1 (acquired prosopagnosic-like). For each group we plotted the distribution of these trial probability values along that 0–1 continuum, as a density plot. We then compared these distributions between the three groups, asking whether the trials of the developmental prosopagnosic group resembled more those of the acquired prosopagnosic group or the trials of the control group. This showed that the developmental prosopagnosia data more closely resembled that of the control group than that of the acquired prosopagnosia group, being only slightly shifted to the right of the control distribution (Figure 9).

**Figure 10:**
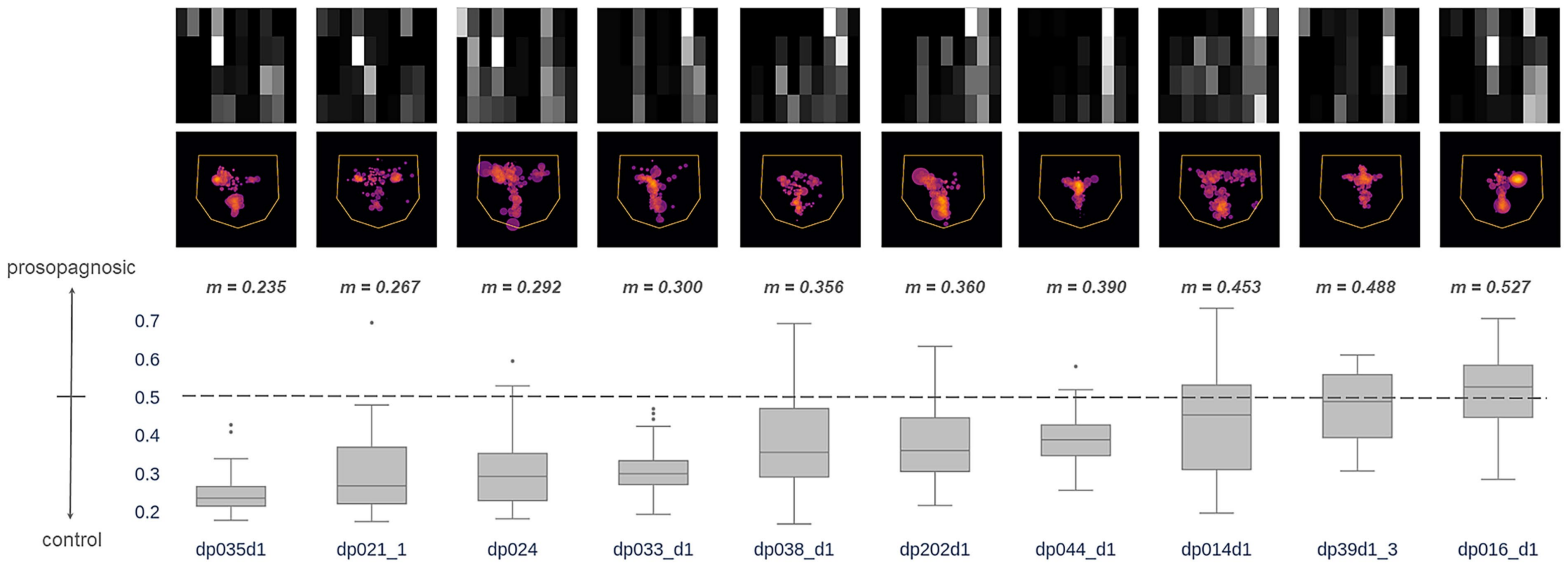
Section 3, individual results for developmental prosopagnosia. The images superimpose the first 4 fixations from all trials for each of the 10 subjects, as done for acquired prosopagnosia in Figure 5. The box plots show the distribution of predictions from the *Hybrid* Model for each subject’s trials using their first 4 fixations (plotting conventions as explained in Figure 5). Subjects are sorted by median prediction from the *Hybrid* Model. As in Figure 5, subjects on the left have fixation patterns that are closer to the model’s understanding of a control subject, and those to the right have characteristics that match the model’s understanding of the scanning of acquired prosopagnosia. All but one subject had a median predicted value lower than 0.5 (i.e., closer to controls than to acquired prosopagnosia).

For individual subjects, we examined how the model characterized the scanpaths of each of the 10 subjects with developmental prosopagnosia (Figure 10). This showed a range of median predictions, with only one subject (DP016) appearing slightly more like acquired prosopagnosia (i.e., median prediction ≥ 0.5) and the rest having median predictions below 0.5, and therefore biased more toward controls.

How did this single-subject analysis contrast between the cohorts? For the control group, the mean single-subject prediction was 0.31 (s.d. 0.11), whereas it was 0.49 (s.d. 0.28) for acquired prosopagnosia and 0.37 (s.d. 0.10) for developmental prosopagnosia. The difference between acquired prosopagnosia and controls was significant [t_(22)_ = 2.75, *p* < 0.012], whereas the difference between developmental prosopagnosia and controls was not [t_(24)_ = 1.23, *p* = 0.23].

### Relation to individual behavioral performance

We asked two questions. First, from the results of sections 1 and 3, are those with more severe deficits more likely to show scanpaths typical of acquired prosopagnosia? If so, there may be a negative correlation between face recognition accuracy and a scanpath predicting a higher probability—i.e., more typical of acquired prosopagnosia. We analyzed all 18 acquired and developmental prosopagnosic subjects together, using the probabilities of the classifier trained to discriminate acquired prosopagnosia from controls. Scanpath probability showed a trend to a negative correlation with their discriminative accuracy (d’) during the experiment [r = −0.42, *F* (1,17) = 3.55, *p* = 0.076], as well as a similar trend with their Cambridge Face Memory Test scores [r = −0.44, *F* (1,17) = 4.02, *p* = 0.061].

Second, we asked whether the dispersal of fixations seen in developmental prosopagnosia in section 2 was related to recognition performance. Rather than inverse correlations, we found a trend to a positive correlation with d’ in developmental prosopagnosia (r = 0.57, *p* = 0.064). Thus, while the developmental prosopagnosic group had more dispersed scanpaths than controls, within this group those with more dispersed scanpaths tended to recognize faces better.

### Scanpath correlations with performance in control subjects

Since the face perception of healthy subjects falls on a continuum, does their recognition accuracy correlate with any of the scanning variables that distinguish prosopagnosic from control subjects? The dispersion index measured over the first 16 fixations was not related to d’ in the control group (r = 0.26, *p* = 0.25). We also assessed whether recognition accuracy was related to the left: right and eye: mouth indices for the first 4 fixations, which were useful features in distinguishing acquired prosopagnosia from control subjects. While there was no relation between the eye: mouth index and d’ (r = 0.007, *p* = 0.97), there was a trend to an inverse correlation between the left: right index and d’ (r = −0.41, *p* = 0.066). However, we caution that these are *post-hoc* analyses on a small sample and can only be regarded as preliminary explorations.

## Discussion

We explored a deep learning approach for studying the face scanning patterns of prosopagnosic subjects. For acquired prosopagnosia in Section 1, the highest accuracy (80%) in classifying trials was achieved with the first four fixations, with acquired prosopagnosic trials anomalously biased toward the mouth and the eye in right space. In Section 2, optimal classification accuracy for developmental prosopagnosia was lower (69%) and required 16 fixations, with a more peripheral scattering of fixations typifying developmental prosopagnosia. Our third section showed that the scanpaths of developmental prosopagnosic subjects resembled those of controls more than those of subjects with acquired prosopagnosia.

The fixations in scanpaths likely target the most useful areas of a visual stimulus for a perceptual determination (57, 58). For faces, these may be guided by an internal schema that maps the discriminative utility of various regions of the face, creating a hierarchy for facial features (59), though this may vary with the perceptual task (12). For identity processing, the upper face and the eyes play a key role (60). Accordingly, the scanpaths of healthy subjects show a preference for the upper face and eyes when they are identifying faces (3, 6, 61). In addition, healthy subjects fixate the eye in their left hemifield preferentially (62, 63), whether the face is upright or inverted (3). Like the left visual-field superiority for face processing in tachistoscopic studies (64), a left fixation bias has been attributed to a right hemispheric dominance for face processing.

However, scanpaths can be highly variable. Facial scanpaths can show substantial and durable between-subject idiosyncrasies (27, 28), while the scanpaths used by a given subject can differ between different faces, or between different trials using the same face (7, 29), the latter sometimes as a function of task (6). Hence the facial scanpath biases seen in healthy subjects emerge primarily as tendencies in data amassed from many trials in many subjects. Likewise the differences we observed related to prosopagnosia emerged from a deep learning approach using relatively large numbers of trials from groups of subjects, rather than single cases. Our results showed that both eye/mouth and right/left biases distinguish control scanpaths from the scanpaths of acquired prosopagnosia within the first few fixations. In contrast, these biases do not discriminate between control and developmental prosopagnosic scanpaths. Rather, the centrality of fixations is the differentiating factor in this latter comparison. This recalls another finding, that an emphasis on central facial regions is a characteristic fixation pattern of healthy individuals (65).

There are few studies of face scanning in acquired prosopagnosia, reflecting the rarity of this condition. The first two found no difference between three patients and controls (30, 31). A third examined scanning while two patients identified ambiguous morphed faces (19). One patient showed a normal feature hierarchy but lacked the typical left-side bias, while the second fixated external features and the lower face more, and even showed a right-side bias. Of two other subjects making familiarity judgments, PS fixated first the mouth then the eye in left space (20), while SC looked more at external features (21). These heterogeneous results accord with our analysis of individual prosopagnosic subjects, which showed that scanning was more anomalous in some subjects than in others (Figure 5).

Of note, the presence of a hemifield defect did not determine the frequency with which a subject showed this anomalous acquired prosopagnosic pattern, nor did the location of the lesion. Hence the abnormal pattern identified by the classifier is a marker for acquired prosopagnosia in general, rather than some associated field loss or anatomic feature, and, when combined with our developmental prosopagnosic data, may show some correlation with the severity of the recognition deficit.

For developmental prosopagnosia, others found that fixations in the first 7 s were more dispersed in four subjects making familiarity judgments (22). Reduced fixations on the eyes was reported in two family members (25) and in subject K (66), who also fixated the external features more. A study of 10 subjects did not find a scanning difference between internal and external features, though reduced scanning of the internal features did correlate with the severity of the face recognition deficit (23). This study also found fewer fixations of the eyes and more of the mouth at a group level. A heatmap analysis of 12 subjects showed a preserved emphasis on the eyes but with more fixations of the nose and mouth than controls (24). Our results agree most with the two studies (22, 66) that identified a peripherally dispersed fixation pattern as being characteristic of developmental prosopagnosia (Figure 7). Interestingly, this pattern may be associated with difficult perceptual performance in healthy subjects too, who show more dispersed fixations when contrast-reversed images are used to make visual processing more challenging (67).

One of the strengths of our study was the ability to compare a data-driven approach with hypothesis-driven approaches, given that the same data were previously analyzed using the latter (26, 44). Pancaroglu et al. (44) found that most of the patients with acquired prosopagnosia retained the normal bias for the eyes, with the exceptions of BAT1 and BIOT2, though at the group level there was a lower eye/mouth fixation ratio. Lee et al. (26) found no difference in eye/mouth ratios in either acquired or developmental prosopagnosic cohorts, but a bias to more peripheral facial regions for acquired prosopagnosia subjects with occipitotemporal lesions. Notably, this peripheral shift was not found in developmental prosopagnosia, though the current study shows that it is present in the early fixations of this group. Neither of these two prior studies searched for left/right differences, as these were not hypothesized. Thus, the current approach (a) revealed biases that had been sought but difficult to find with the prior methods, (b) allowed non-predicted findings to emerge, and (c) clarified differences between acquired and developmental prosopagnosia.

One methodologic factor that may have played a key role in the current study was the identification of the optimal number of fixations to use. If fixations on an image are directed and sequenced to maximize the rate of accrual of diagnostic information, then differences between those with normal and anomalous recognition skills may be more apparent in earlier fixations. For faces, healthy subjects only require 2–5 fixations to make familiarity judgments (68, 69). These early fixations already show the characteristic normal biases favoring the left hemifield (70) and the eyes (68), as well as influences of familiarity (62). Some case reports have even asked whether prosopagnosic subjects differ from controls in just the first fixation. For acquired prosopagnosia, SB did not (30), but PS looked at the mouth the most (20). For developmental prosopagnosia, the father and son pair of LG and RG rarely fixated the eyes with their first glance (25).

Our third section asked whether developmental prosopagnosic subjects scanned faces more like control or acquired prosopagnosic subjects. At a group level the distribution of their trials resembled a slightly right-shifted version of the distribution of trials from controls (Figure 9). At an individual level, most developmental subjects had scanpaths that on average resembled more those of controls (Figure 10). However, the individual results fell along a spectrum and we cannot exclude the possibility of heterogeneity within the cohort with developmental prosopagnosia. Heterogeneity is a potential issue given that the diagnosis of developmental prosopagnosia is currently based on behavioral and statistical criteria, without definite imaging or genetic biomarkers (15). On the whole, though, the results suggest that the internal representations of faces—facial schemata—that guide scanning behavior are less disrupted in developmental prosopagnosia than in acquired prosopagnosia. Such results question how closely developmental prosopagnosia parallels the acquired form.^2^ These results are relevant to the debate as to whether developmental prosopagnosia represents simply the low end of a normal spectrum of perceptual ability, or a pathologic entity resulting from aberrant development (15). While our results do not prove the point, the fact that the facial scanpaths of developmental prosopagnosic resemble control scanpaths more may be more consistent with the normal-spectrum concept.

To summarize, the current deep learning approach advanced upon the prior studies (26, 44) by showing, first, that optimal differentiation of acquired prosopagnosia from controls required only four fixations, while 16 fixations was needed for discriminating the scanpaths of developmental prosopagnosic subjects from controls. Hence acquired prosopagnosic scanpaths show anomalies that are apparent very early with good discriminative power (AUC 80%), whereas differences in developmental prosopagnosia are more subtle, requiring more fixations to discern, with less discriminative power (AUC 69%). Second, within these early samples of fixations, the classifier detected a tendency for acquired prosopagnosic scanpaths to focus less on the eyes and more on the mouth compared to controls, a difference that had been hypothesized but difficult to show using the entire sample of fixations (26, 44). Third, it revealed a bias toward the eye on the right side of the image in acquired prosopagnosia, which had not been examined on an *a priori* basis in those prior studies. Fourth it showed that these two biases are not characteristic of developmental prosopagnosia, where scanning resembled a more dispersed version of control scanpaths. In contrast, the prior study had not been able to show any difference between developmental prosopagnosic subjects and controls (26). Nevertheless, for our fifth key finding, the final section showed that developmental prosopagnosic scanpaths are more similar to those of controls than to those of subjects with acquired prosopagnosia.

There are three implications of the current results. First, the anomalous scanpaths of acquired prosopagnosia suggest disruption of a facial schema that shows where the most useful information about facial identity is located, the result being that they scan the mouth and the eyes nearly equally while controls scan the eyes more. Second, given that the right and left eyes have similar utility for identity judgments (12), the shift away from the eye on the left side of the image may be related less to distorted information processing and more to loss of the right hemispheric dominance for face identification, which is consistent with the fact that all of our acquired prosopagnosic subjects had structural lesions in the right hemisphere. Third, and consistent with the prior conclusion (26), the fact that these anomalous biases are not seen in developmental prosopagnosic subjects suggests that the latter still possess a relatively normal facial schema.

The dispersion seen in developmental prosopagnosic scanpaths has several possible explanations. One is that the facial schema is more degraded and less precise, or its use in directing attention and fixations is less accurate. Alternatively, given the trend to more dispersed scanpaths being associated with better face discrimination in these subjects, it may represent a useful strategic response to widen the field of sampling when a perceptual system is faced with diagnostic uncertainty. Relevant to this point, visual search tasks analyzed with linear ballistic accumulator models show that developmental prosopagnosia is characterized by normal rates of information accumulation but higher evidence thresholds for reaching a decision about face identity (71). Difficulty in reaching a threshold may spur broader stimulus sampling, though still guided by an intact facial schema in developmental prosopagnosic subjects, and the additional evidence obtained by this change in sampling may support more accurate face recognition in this group.

The main limitation of our study is the small dataset. The performance of deep learning models improves with more training data. However, acquired prosopagnosia is a rare condition. The size of the current cohort was achieved only by recruiting across the North American continent, and it represents most subjects of the largest such group examined in recent times. Developmental prosopagnosia may be more common (72) and cohorts of a similar size to ours have been reported in prior work (23, 24). It would be of interest to replicate our results in additional groups of developmental prosopagnosic subjects, particularly given the possibility of heterogeneity. A second caveat is that our results may be culturally specific. Our prosopagnosic and control subjects are all of white ancestry. Eye movement studies have found that, while such subjects fixate the eyes and mouth, east Asians fixate the nose at the center of the face (45), while the initial fixations of black subjects also fixate the nasal region more (46).

To our knowledge, this is the first use of a machine learning approach to study face scanning in prosopagnosia. Our approach identified the optimal segments of the scanpath for classification, as opposed to using all fixations. The end-to-end nature of deep learning eliminated the need for manual feature addition, which is useful when group characteristics are not fully understood. The advantages of this approach were made apparent by comparing its results directly with prior analyses of the same data using a traditional hypothesis-driven approach (26, 44). Whereas the prior work suggested only a modest preference for the lower face in acquired prosopagnosia, the current approach confirmed this, showed that there was also an anomalous bias to the right side of facial images, and revealed that these biases were already apparent in the first four fixations. Furthermore, this method revealed that the scanpaths of developmental prosopagnosia did not mirror those of acquired prosopagnosia, showing instead a dispersal to more peripheral regions, and overall resembling control scanpaths more than the anomalous scanpaths of acquired prosopagnosia.

## Data Availability

The raw data supporting the conclusions of this article will be made available by the authors, without undue reservation. Requests to access these datasets should be directed to jasonbarton@shaw.ca.

## Footnotes

^1^ https://kdef.se/

^2^Similar concerns raised for other reasons have led some to propose the unwieldy name of “prosopdysgnosia” for the developmental form (73).

## Supplementary material

SUPPLEMENTARY Figure 1 This scatterplot shows the sensitivity (d’) and bias (c) for each participant in the behavioural face recognition protocol. Black filled circles denote individuals with acquire prosopagnosia, open circles denote individuals with developmental prosopagnosia, and light grey circles denote controls.

## Appendix. Features used in the baseline model

### Features added based on previous literature

**Table:**

Feature	Description and rationale
Central Duration	Time spent scanning central regions (eyes, nose and mouth) during the first *m* fixations of a trial. The scanning of healthy subjects is biased toward the center of the face (3).
Peripheral Duration	Time spent scanning peripheral regions (eyebrows, cheeks, forehead, chin) during the first *m* fixations.
Eyes Duration	Time spent scanning the eyes during the first *m* fixations. Healthy individuals fixate the eyes more than the nose or mouth (3).
Nose/Mouth Duration	Time spent scanning the nose and mouth during the first *m* fixations. Prior work suggests that prosopagnosic patients may be biased toward the lower central regions (73)
Phase of Study	An integer indicating the experimental phase and faces. 1. *Learning Phase*. the subject is studying the target faces. 2. T*arget Recognition Phase*. The subject is shown a face they had seen in the learning phase. 3. *Distractor Recognition Phase*. The subject is viewing a face that had not been seen in the learning phase. Prior reports have noted that familiarization can alter the scanning of faces (74).

### Features added based on statistical analysis

**Table:**

Feature	Description
Mean fixation duration	Average duration of the first *m* fixations of a trial. If information acquisition is degraded in prosopagnosia, subjects may require more time to obtain diagnostic information during each fixation.
Fixation duration variance	Variance of the durations of the first *m* fixations of a trial. Information acquisition may be noisy in prosopagnosia, resulting in more variable rates of diagnostic data accrual during fixations.

### Features added based on our assumptions

**Table:**

Feature	Description
Total trial duration	Total duration of a trial, including all fixations. Subjects with difficulty recognizing faces may take more time and use more fixations to view faces that they are attempting to learn or recall.
Total duration	The total duration of a trial’s first *m* fixations.
First fixation duration eyebrows	Time spent scanning each of these ROIs during the first fixation. These features are added to highlight the initial time spent scanning the peripheral regions by different groups.
First fixation duration forehead	
First fixation duration cheeks	
First fixation duration chin	
