# Supplementary figures and images for: Scanning faces: a deep learning approach to studying eye movements in prosopagnosia
Source: Front Neurol. 2025 Sep 10;16:1616509. doi: 10.3389/fneur.2025.1616509 (PMC12459115; doi:10.3389/fneur.2025.1616509)

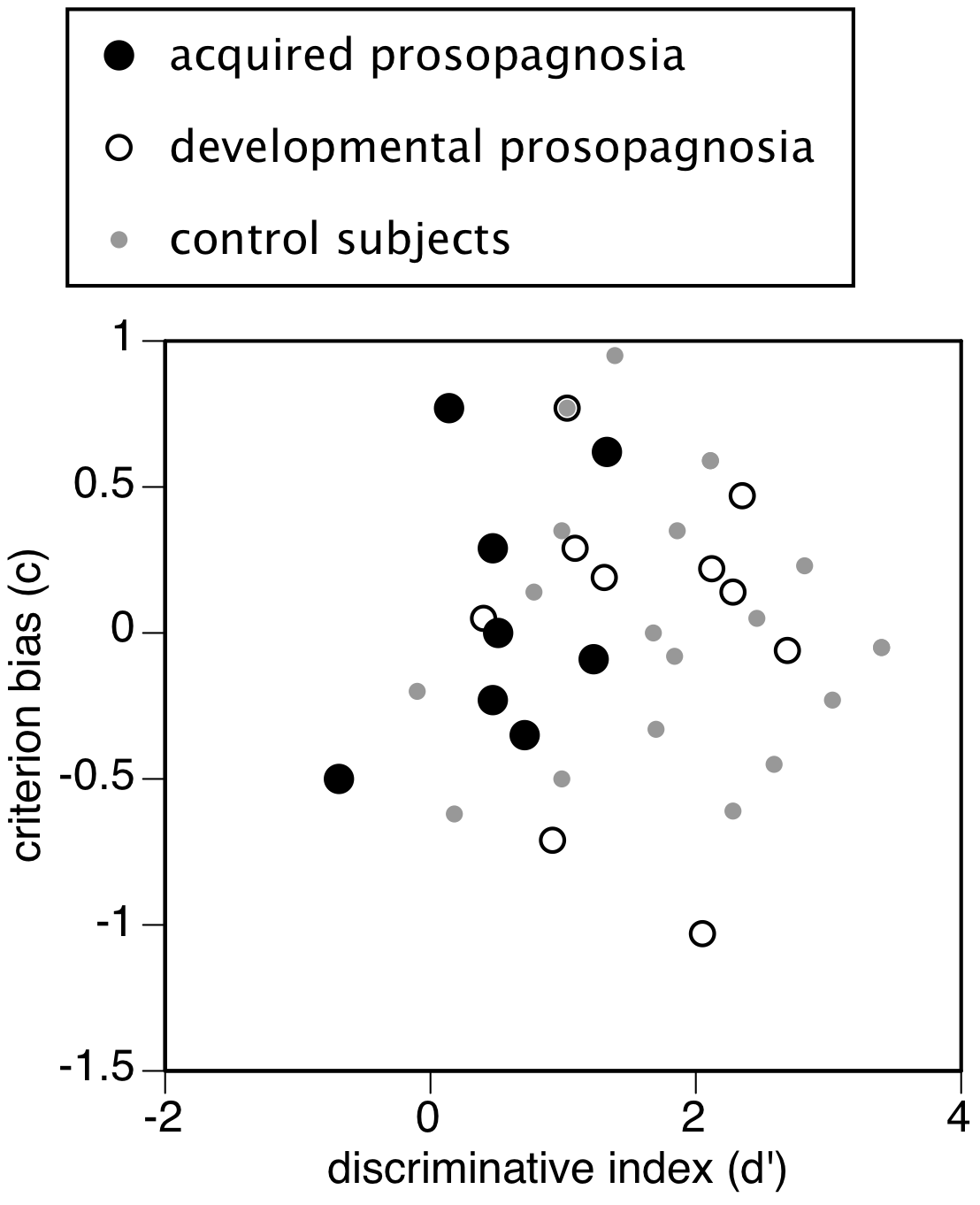

Supplement: SUPPLEMENTARY Figure 1 — This scatterplot shows the sensitivity (d’) and bias (c) for each participant in the behavioural face recognition protocol. Black filled circles denote individuals with acquire prosopagnosia, open circles denote individuals with developmental prosopagnosia, and light grey circles denote controls. [file Image_1.tif]
